# Pulse-Driven Magnetoimpedance Sensor Detection of Cardiac Magnetic Activity

**DOI:** 10.1371/journal.pone.0025834

**Published:** 2011-10-12

**Authors:** Shinsuke Nakayama, Kenta Sawamura, Kaneo Mohri, Tsuyoshi Uchiyama

**Affiliations:** 1 Department of Cell Physiology, Nagoya University Graduate School of Medicine, Nagoya, Japan; 2 Department of Electronics, Nagoya University of Graduate School of Engineering, Nagoya, Japan; National Microelectronics Center, Spain

## Abstract

This study sought to establish a convenient method for detecting biomagnetic activity in the heart. Electrical activity of the heart simultaneously induces a magnetic field. Detection of this magnetic activity will enable non-contact, noninvasive evaluation to be made. We improved the sensitivity of a pulse-driven magnetoimpedance (PMI) sensor, which is used as an electric compass in mobile phones and as a motion sensor of the operation handle in computer games, toward a pico-Tesla (pT) level, and measured magnetic fields on the surface of the thoracic wall in humans. The changes in magnetic field detected by this sensor synchronized with the electric activity of the electrocardiogram (ECG). The shape of the magnetic wave was largely altered by shifting the sensor position within 20 mm in parallel and/or perpendicular to the thoracic wall. The magnetic activity was maximal in the 4th intercostals near the center of the sterna. Furthermore, averaging the magnetic activity at 15 mm in the distance between the thoracic wall and the sensor demonstrated magnetic waves mimicking the P wave and QRS complex. The present study shows the application of PMI sensor in detecting cardiac magnetic activity in several healthy subjects, and suggests future applications of this technology in medicine and biology.

## Introduction

Electrical activity of the heart simultaneously induces a magnetic field. Detection of this magnetic activity will enable non-contact, noninvasive evaluation to be made. So far, magnetocardiography (MCG) has been performed by using a superconducting quantum interference device (SQUID), and applied to the diagnosis of ischaemic cardiac diseases and arrhythmias, including the fetal heart [1]–[3]. This technology however, requires extremely low temperatures to maintain superconductivity, and is also often equipped with a magnetic shield to reduce environmental magnetic noise [4], [5]. The total system is therefore too large and expensive for personal and single laboratory use.

In the present study, we thus employed a pulse-driven magnetoimpedance (PMI) sensor [6], [7], which is used as an electric compass in mobile phones and as a motion sensor of the operation handle in computer games, to measure magnetic activity in the human heart. The sensor head consists of a pickup coil with a small CoFeSiB amorphous wire as a magnetic core. In this sensor system, a complementary metal oxide semiconductor (CMOS) IC repeatedly triggers the supply of an electric current to the amorphous wire in order to set the direction of electron spin along the wire axis. In doing so, the sensitivity in response to external magnetic field dramatically increases to a pico-Tesla (pT) level [8], [9]. At the same time the CMOS IC triggers a sample-and-hold circuit to measure the off-diagonal voltage response in the pickup coil [9]. An operation interval of 1 µs is fast enough to achieve quasi-real time recordings to follow biological activities. Compared to ordinary one, the sensitivity of the PMI sensor used in the present study has been improved by increasing the turns of the pickup coil and adjusting the circuit and parameters for electric pulse excitation according to theoretical optimization [7], [8].

Since this new magnetic field detection system is constructed solely from ordinary electromagnetic materials and operates at body temperature, it is possible to use the detector very close to living systems. Here we show the PMI sensor system has successfully detected local magnetic activity on the surface of the human chest, and synchronizes with cardiac electric activity. Furthermore, averaging local magnetic signals detected by the PMI sensor system has provided magnetic waves mimicking those previously measured by SQUID sensors.

## Materials and Methods

### Participants

Five healthy subjects (male, 20–50 years-old) volunteered for the present study to measure cardiac magnetic activity. During measurements, participants wore a cotton shirt less than 2 mm thick, and sat on a chair. Procedures of magnetic measurements were approved by the Ethics Committee of Nagoya University Graduate School of Medicine. Written informed consent was obtained from all participants. This study complies with the Declaration of Helsinki.

### Magnetocardiogram

**Figure 1:**
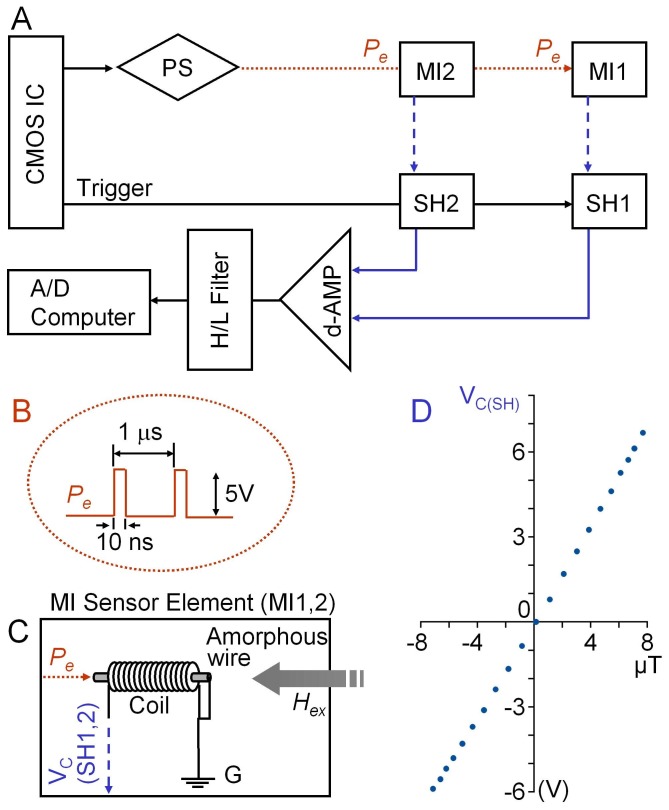
Schematic diagram of a PMI sensor system. A) MI sensor elements (sensor head: MI1, MI2) receive driving electric pulses (*P_e_*) from a power supply (PS) triggered by a CMOS IC. At the same time, the CMOS IC triggers sample-and-hold detectors (SH1, SH2) to measure the voltage of the pickup coils in MI elements. The operation amplifier (d-AMP) differentiates the voltage in SH2 from that in SH1 to detect biomagnetic activity measured close to MI1. The biomagnetic activity is recorded by a 14bit A/D converter via high and low cut electric filters (high cut 40 Hz; low cut 0.3 Hz). B) Excitation electric pulses (*P_e_*) of 10 ns in duration and 5V in amplitude are applied at 1 µs intervals. C) Each MI sensor elements consists of an amorphous wire (30 µm in diameter, 5 mm in length) surrounded by a pickup coil (1 mm diameter, 500 turns), and detects magnetic signals in the same direction as the amorphous wire. D) Voltage at sample-and-hold unit (V_C(SH)_) linearly changes with a very high sensitivity of ∼88 µV/nT.

Cardiac magnetic activity was measured using a lab-made magnetocardiogram (MCG) system equipped with a pair of pulse-driven MI sensors. Figure 1A shows a block diagram of the MCG system used in the present study [6], [7]. A CMOS IC triggers a power supply (PS) that applies electric pulses of 5V (10 ns) to a pair of MI sensor elements (MI1, MI2). The same CMOS IC simultaneously triggers a pair of sample-and-hold circuits (SH1, SH2) to detect the induction voltage of the pickup coils in MI1 and MI2. As shown in Fig. 1B, PS repeatedly provided electric pulses of 10 ns in duration and 5V in amplitude at 1 µs intervals. Panel in Fig. 1C shows each MI sensor pickup coil (MI1, MI2) which consists of a CoFeSiB amorphous wire (30 µm in diameter, 5 mm in length) and a transducer coil (1 mm diameter, 500 turns) surrounding the magnet wire. This PMI MCG system achieved a very high sensitivity of ∼88 µV/nT (∼0.11V/A/m) and an almost completely linear voltage conversion of the magnetic field within the range of ±7.5 A/m (Fig. 1D) [9]. The external magnetic field magnetizes the amorphous wire in the axial direction, while the excitation electric pulse magnetizes the wire in the circular direction. Since our PMI system employs pickup coils to detect the magnetization in the amorphous wire, unlike vibrator-type MI sensors, it is considered that the noise level in the coil does not increase until sizeable amplitude of electric current applied. Also as the circuit of the amorphous wire excitation has the resistance of ∼30 Ω, the repetitive application of electric pulse produces the peak amplitude of ∼170 mA in each pulse to increase resolution (sufficiently suppress the magnetic noise). On the other hand, the inhibitory effect of bias direct current (low frequency harmonics) on sensitivity is still low [8] because of the short duration in electric pulse, i.e. 10 ns.

During measurements, the MI1 sensor head (element) was placed perpendicularly on the chest of each subject, while the other sensor head, MI2, was 50 mm behind MI1 in the same direction of the amorphous wire of MI1 (Figure S1). MI1 detected changes in the magnetic activity from the heart along with environmental magnetic noise, while MI2 mainly detected the latter. The position of magnetic measurement (MI1 sensor head) was assigned using V_1_ and V_2_ in ECG (4th intercostals on the right and left lateral sternal lines, respectively). An operation amplifier was used to differentiate MI2 from MI1 magnetic activity (induction voltage of SH2 from SH1) to cancel environmental magnetic noise, i.e. a gradiometer. After the operation amplifier, high and low cut filters (H/L filter: 40 and 0.3 Hz, respectively) were applied, and resultant voltage signals were stored in a computer through an A/D (14 or 16 bit) converter. Positive magnetic signals in figures represent magnetization toward the MI1 sensor head.

### Electrocardiogram

**Figure 2:**
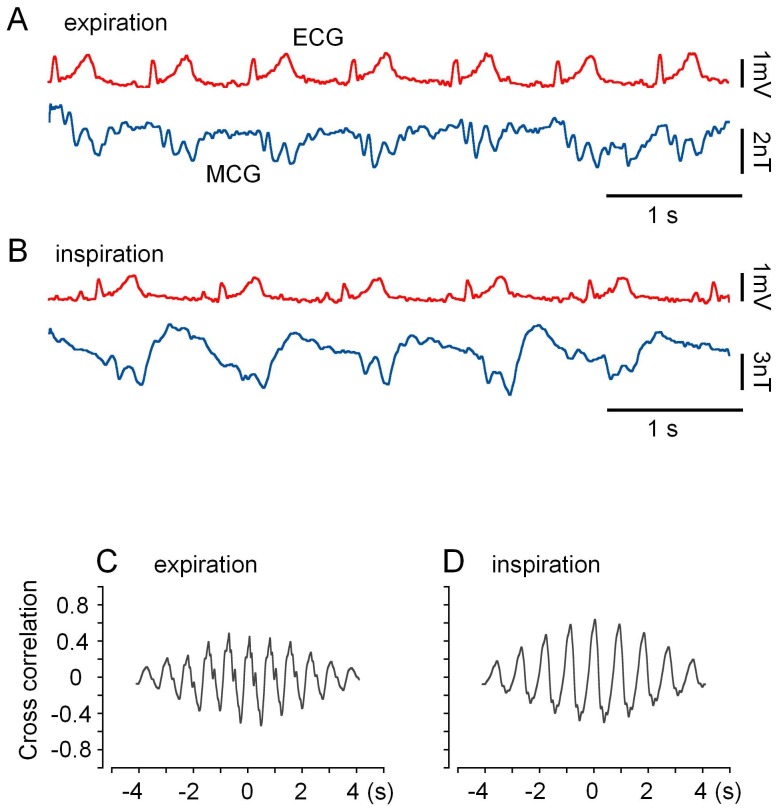
Magnetic activity recorded near V_2_ on the surface of the human chest. Magnetic activity was measured together with the limb lead I ECG during deep expiration (A) and inspiration states (B). The distance between the MI1 sensor head and the chest wall (*d*) was ∼2 mm. The magnetization toward the sensor head is positive. The cross-correlation functions between ECG and MCG in (C) and (D) correspond to (A) and (B), respectively.

**Figure 3:**
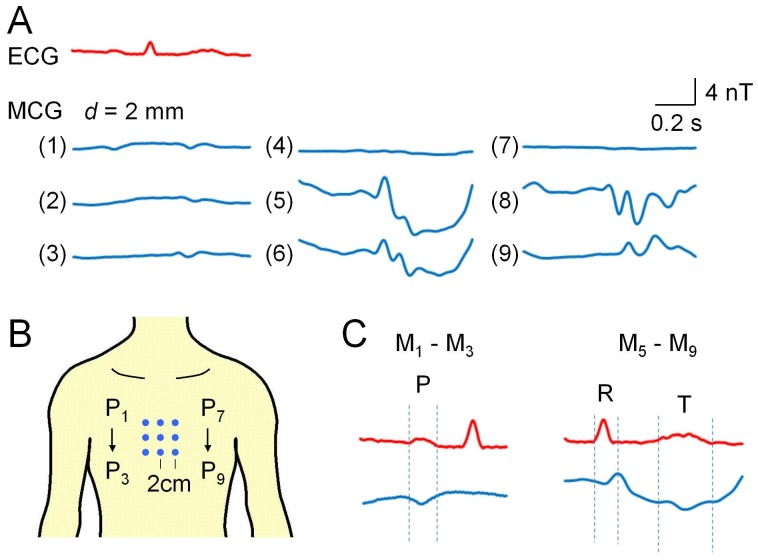
Spatial distribution of magnetic activity. Magnetic activity (A) measured at 3×3 positions on the surface of the chest (B). P_2_ and P_8_ in this measurement correspond to V_1_ and V_2_ positions in ECG, respectively. The distance between the sensor head and the chest wall: (*d*) = 2 mm. Limb lead I ECG was simultaneously measured to average magnetic signals during three consequent cardiac cycles. In C, subtractions of M_1_ – M_3_ and M_5_ – M_9_ are shown. Note magnetic waves corresponding to ECG waves, i.e. P wave, QRS complex and T wave.

**Figure 4:**
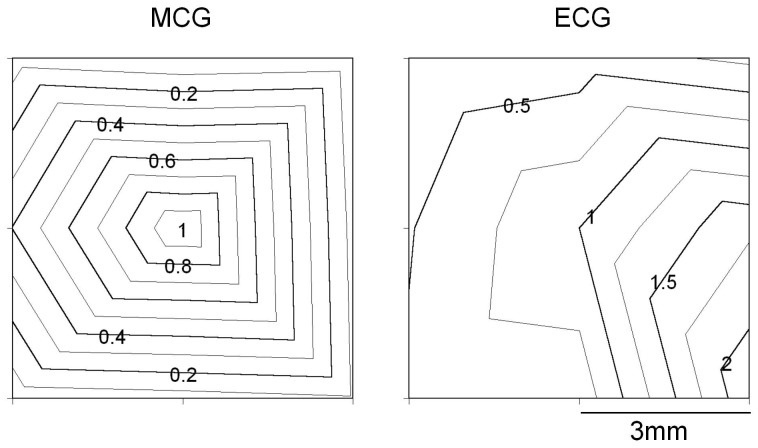
Spatial distribution of magnetic and electric power. Magnetic and electric (ECG) activities were separately recorded from a healthy subject at 3×3 matrix points on the chest. P_5_ was the center between V_1_ and V_2_ positions in the ECG. The distance between each adjacent measurement position was ∼3 cm. After Fourier transformation, the power of 1–40 Hz was plotted as a contour map. The power value was normalized by that of P_5_ (magnetic measurement), and by that of P_9_ (electric measurement).

**Figure 5:**
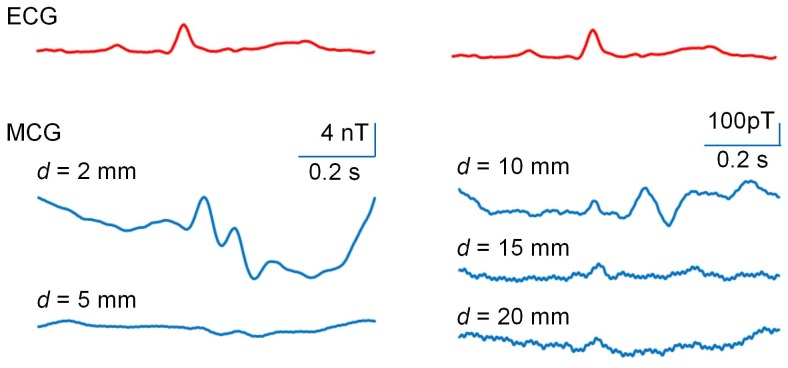
Changes in magnetic wave depend upon the distance between the sensor head and the chest (*d*). Magnetic activity (blue) was measured at P_5_ with *d*  =  5, 10, 15, and 20 mm. To average magnetic signals, limb lead I ECG (red) was simultaneously recorded. Each magnetic waves shown is an average obtained during three consequent cardiac cycles.

**Figure 6:**
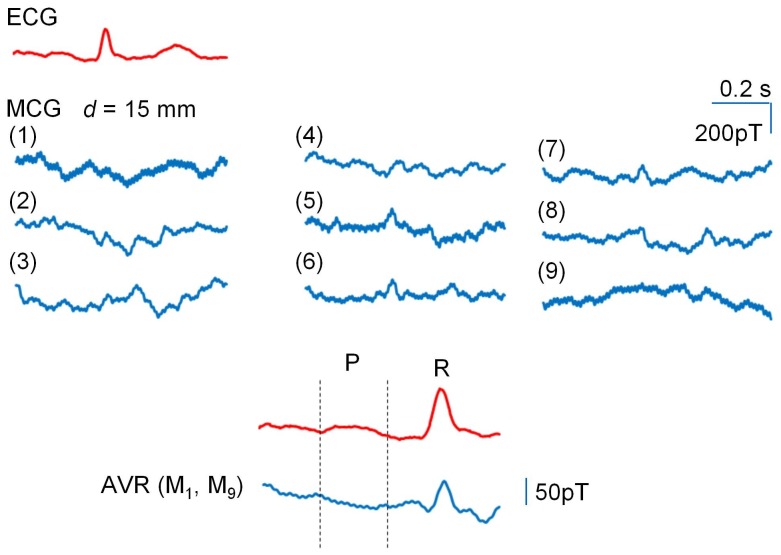
Magnetic waves measured with a greater distance of *d*  =  15 mm. Upper panel) Spatial distribution of magnetic activity at 3 x 3 positions as shown in Fig. 5: The distance between each adjacent measurement position was ∼2 cm. Lower panel) An averaged magnetic wave constructed from all magnetic waves measured at 3 x 3 matrix points. Note that the P wave and QRS complex are relatively easily assigned.

Limb lead I of the electrocardiogram (ECG) was monitored along with PMI MCG, and used for cross-correlation analysis against the MCG signals in Fig 2. In Fig. 3, the R wave of ECG was used as a time reference of the cardiac cycle for arithmetic calculation between magnetic signals. In Fig. 4, chest lead unipolar ECG was separately carried out in order to compare the distribution of the power between magnetic and electric fields. In Figs. 5 and 6, again, the R wave of ECG was used as a time reference of the cardiac cycle.

### Data analysis

To compare our recordings of local magnetic activity with previous MCG recordings using the SQUID system, we integrated local magnetic signals using the R wave of limb lead ECG as a marker. Also, some time-domain data was converted into frequency-domain data (power spectrum), and a digital filter was applied using commercial software (DIADEM: National Instruments).

## Results

### Cardiac magnetic activity measured using PMI

A PMI sensor system (Fig. 1) was used to measure cardiac magnetic activity in healthy subjects sitting on a chair in an experimental room without any magnetic shield equipment. The PMI sensor head, MI1 (the detector of cardiac magnetic activity: see Methods) was perpendicularly placed, approximately 2 mm apart from the thoracic wall. The other PMI sensor head MI2 was 50 mm behind MI1 in the same direction, in order to cancel the environmental magnetic noise.

Figure 2 shows an example of cardiac magnetic activity near V_2_ on the surface of the chest in a healthy subject. Cardiac electric activity (ECG) was simultaneously monitored in limb lead I. Respiratory activity largely affected the baseline of recordings and the shape of magnetic waves, probably by changing the location of the heart in the thoracic cavity, therefore, measurements were carried out under breath-held conditions. Traces in Fig. 2A and 2B were obtained during breath-held deep expiration and inspiration, respectively. However, cross-correlation analysis (C and D) showed that the magnetic and electric activities were well synchronized under both conditions. This indicates that the magnetic activity measured on the surface of the chest originated from cardiac electric activity, despite of a large difference in the shape of magnetic waves.

### Distribution of cardiac magnetic activity

Next, we sequentially measured magnetic activity at nine (3×3) matrix points of the chest wall, as shown in Fig. 3. The measurement positions 2 and 8 (P_2_ and P_8_) were near V_1_ and V_2_ in ECG, respectively, and P_5_ was the center between P_2_ and P_8_. The distance between these positions was approximately 2 cm. The set of 3×3 traces in the upper panel shows an example of such a magnetic measurement carried out in another subject under shallow inspiration. Each trace was constructed by averaging three consequent magnetic signals. Unlike ECG, the wave shape of magnetic signal varied largely between the measurement positions on the chest. Namely, large slow waves corresponding to the ST period of ECG was observed in P_5_ and P_6_, while the amplitude of the magnetic signal was smaller than 1 nT in P_4_ and P_7_.

In order to assess the relationship between magnetic and electric activities, we made arithmetic calculations between magnetic signals as shown in the lower panels of Fig. 3. Subtraction of the magnetic signal in P_3_ from that in P_1_ (M_1_ – M_3_) (lower, left), and M_5_ – M_9_ (lower right) provided magnetic waves corresponding to the P wave, the QRS complex and the T wave.

Figure 4 compares the distribution of power between magnetic and electric fields in the 3×3 matrix points on the chest. Each power distribution is plotted as a contour map. In A, the same magnetic signals shown in Fig. 3B were used, while in B cardiac electric signals separately measured in chest lead were used. The power was maximal near the center (P_5_) in MCG, while it increased progressively toward the bottom left (P_9_) in the ECG. This result indicates that the local magnetic signals measured in the present study are unlikely to reflect a local electric current on the surface of the chest.

### Space-dependency of magnetic waves

To further assess the property of magnetic waves, measurements were carried out by changing the space between the PMI sensor head and the chest wall (*d*) in the P_5_ position. As shown in Fig. 5, as *d* increased, the amplitude of the large magnetic wave during the ST period decreased rapidly, while the magnetic wave corresponding to the QRS complex decreased slowly, or was even more clearly assigned at *d* = 15 and 20 mm.

The upper panel in Fig. 6 shows the spatial distribution of magnetic activity in the 3×3 matrix points at *d* = 15 mm. The amplitude of the magnetic wave was much smaller than that at *d* = 2 mm, and the wave shape was largely altered. The lower panel shows a magnetic signal constructed by averaging all nine magnetic waves measured in the 3×3 points. Of note is that the P wave and QRS complex were more clearly distinguishable than those at *d* = 2 mm.

## Discussion

In the present study in order to measure cardiac magnetic activity, we employed a pulse-driven MI sensor system at body temperature. As shown in Fig. 2, on the surface of the chest in healthy volunteers, changes in the magnetic field were synchronized with cardiac electric activity, simultaneously measured in the limb lead. This result indicates that our PMI sensor system successfully detected cardiac magnetic activity. Furthermore, the spatial distribution of the power of magnetic activity was distinct from that of electric activity recorded in the chest lead. Namely, the magnetic activity power was maximal in P_5_ (in the center of the 3×3 matrix points), while the electric power was maximal in P_9_ (in the bottom left of the matrix points), in good agreement with previous comparisons between chest lead ECG and magnetic activity measured with SQUID sensors [5]. This observation also indicates that the electric field on the surface of the chest does not affect our PMI sensor measurements.

SQUID MCG often estimates magnetic activity by separating the chest with a 4×4 cm grid into 6×6 or 8×8 sections. A pair of gradiometers measures magnetic signals integrating the whole magnetic field of each section, and detects the P wave, the QRS complex and the T wave in each section [5], [10], [11]. On the other hand, magnetic waves measured with PMI sensors largely changed within a small area (∼4×4 cm) on the surface of the chest (Fig. 3). Note that relative amplitude of the P wave to the QRS complex was maximum on the top right (P_1_), namely the chest region close to the atrium. Despite lower sensitivity, PMI sensors appear to achieve MCG with higher spatial resolution, due to their small sensor head amorphous wire (30 µm in diameter and 5 mm in length).

When the PMI sensor head is very close to the chest wall (see Fig. 5, at *d* = 2 mm), a slow magnetic wave is prominent during the ECG ST period. However, this component rapidly decreases as the distance (*d*) increases. Instead, magnetic waves corresponding to the P wave and the QRS complex become clearer. Since PMI sensors act as vector sensors detecting an external magnetic field in the same direction of the amorphous wire, these sensors reflect only limited magnetic signals close to the small sensor head. As the distant from the chest wall increases, the cardiac region provides wider magnetic signals toward the sensor head, presumably depending upon the Cauchy-Lorentz distribution (Figure S2) [12]. It is considered that at *d* = 15–20 mm the PMI sensor detects magnetic activity in a manner that mimics SQUID MCG measurements, because the P wave and the QRS complex are relatively easily assigned in averaged magnetic signals of the 3×3 matrix points (in a 4×4×cm square) measured at *d* = 15 mm (Fig. 6), as seen in previous SQUID sensor measurements. On the other hand, the origin of the slow magnetic wave during the ST period is not clearly indicated, but is suggested to be due to a local current in the anterior wall of the heart and/or thoracic wall itself, because it was rapidly reduced by increasing the distance between the sensor head and the chest wall. Also, some capacitive effect on MI1 detector coil might be involved.

Numerous applications have so far been suggested for MI sensors, for instance, to use as motion detectors in mobile phones, vehicles and robots. In addition to such industrial applications, the present study indicates the direction of future medical and biological applications, because averaging local magnetic signals provides signals similar to those measured with SQUID, which is thought to be reliable in detecting a cardiac magnetic field. Since MI sensors stably work at body temperature [13], this technology is particularly advantageous to biological applications. Future developments would include increasing the sensitivity and developing a multi-channel system. Such improvements would expand the possible applications of the MI sensor into the whole body, including measurement of brain magnetic activity. Use of this type of magnetic sensor with catheter in clinical diagnosis and intervention [14], [15] is also anticipated, for instance, to more precisely characterize any arrhythmias and abnormal conductions, e.g. premature ventricular beat, bundle-branch block, etc. Furthermore, MI sensors could be applicable to stem cell research, i.e. evaluation of electric (magnetic) excitability during differentiation and development of embryonic stem cells [16], [17] and induced-pluripotent stem cells [18]-[20]. It has already been shown that MI sensors can detect magnetic activity accompanied by spontaneous electric activity in isolated muscle tissues [7], [21], [22].

In conclusion, we have shown the successful application of pulse-driven MI sensor in detecting cardiac magnetic activity, and suggest several future applications of this technology.

## Supporting Information

Figure S1 Photo showing PMI sensor measurement of cardiac magnetic field in a participant sitting on a chair. The position of the sensor head (arrow) was adjusted by shifting a plastic mount.
(TIF)

Figure S2PMI sensor measurements with short and long distance between the sensor head and chest wall. Since the PMI sensor is a vector sensor, the spatial detection efficacy (*E*) (spacing loss) of magnetic signals can be expressed as a Lorentzian function [12]:

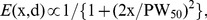
(1)

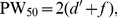
(2)where x and *d'* are the distance from the detection center (projection line from the amorphous wire) and the distance between the sensor head and magnetic signal source, respectively. *f* represents the arctangent parameter (inversely proportional to the maximal slope) of the magnetic signal. PW_50_ indicates the half width of this function.(TIF)
