# Supplementary figures and images for: Pulse-Driven Magnetoimpedance Sensor Detection of Cardiac Magnetic Activity
Source: PLoS One. 2011 Oct 12;6(10):e25834. doi: 10.1371/journal.pone.0025834 (PMC3192116; doi:10.1371/journal.pone.0025834)

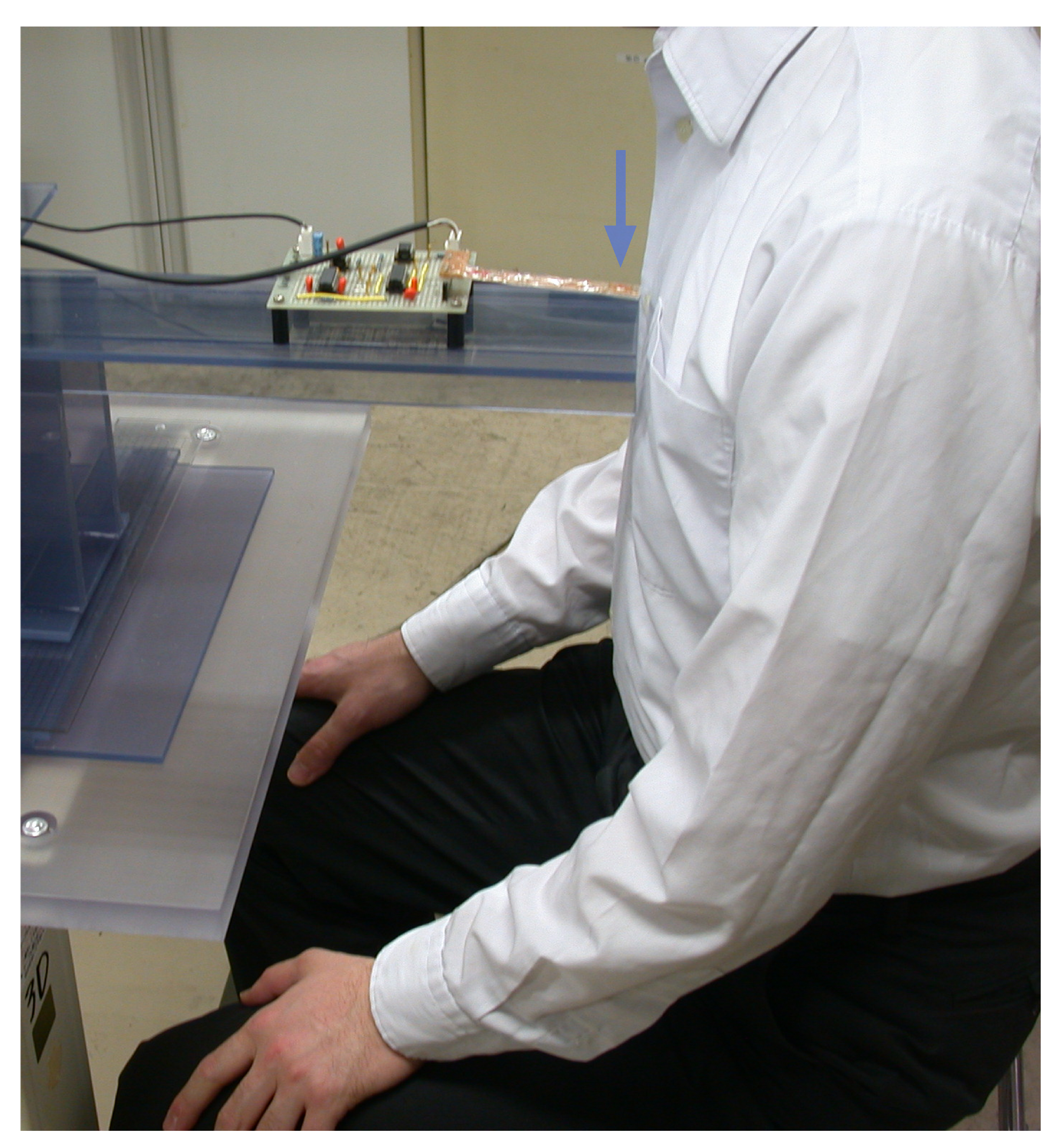

Supplement: Figure S1 — Photo showing PMI sensor measurement of cardiac magnetic field in a participant sitting on a chair. The position of the sensor head (arrow) was adjusted by shifting a plastic mount. (TIF) [file pone.0025834.s001.tif]

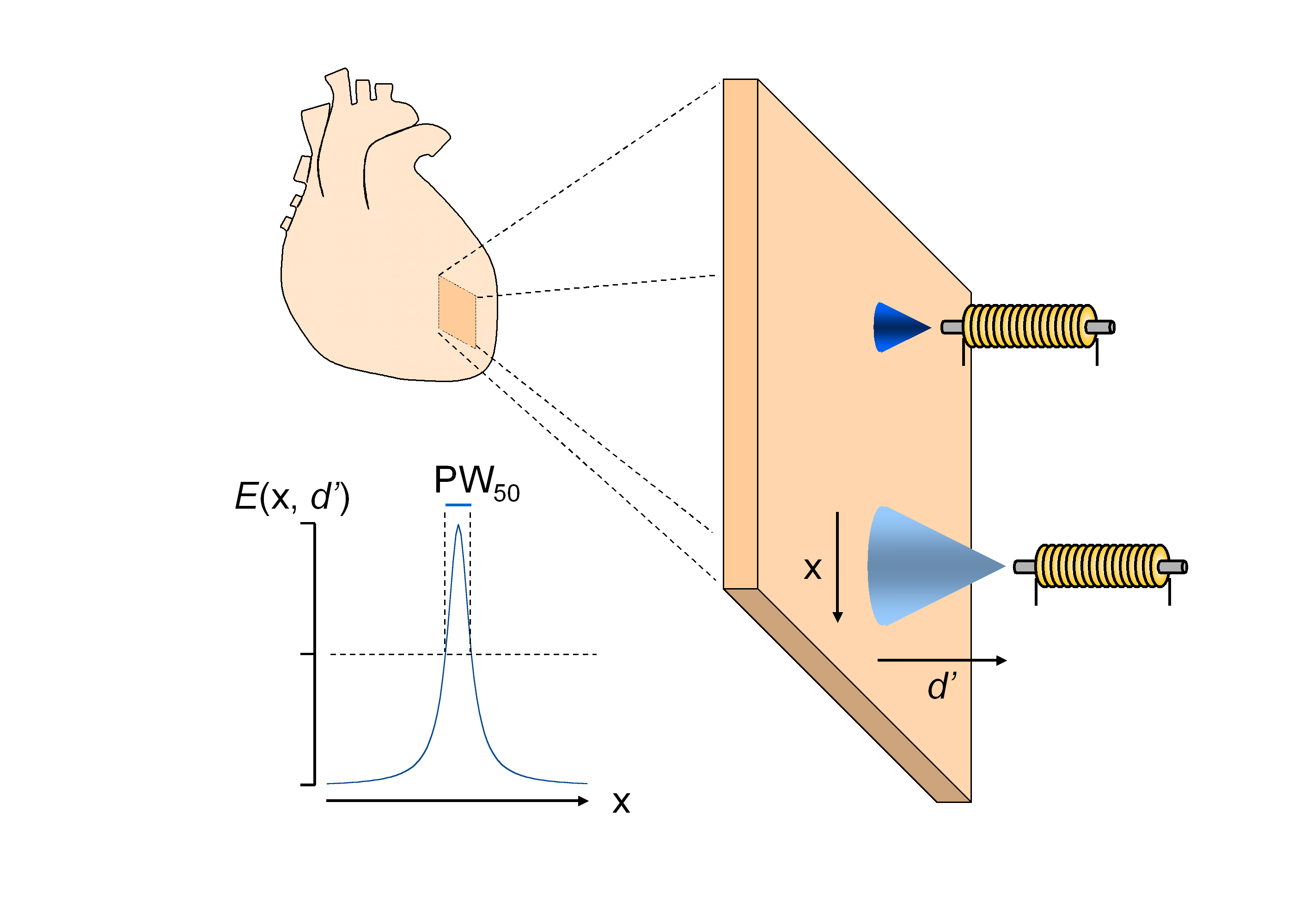

Supplement: Figure S2 — PMI sensor measurements with short and long distance between the sensor head and chest wall. Since the PMI sensor is a vector sensor, the spatial detection efficacy (E) (spacing loss) of magnetic signals can be expressed as a Lorentzian function [12]: (1) (2)where x and d' are the distance from the detection center (projection line from the amorphous wire) and the distance between the sensor head and magnetic signal source, respectively. f represents the arctangent parameter (inversely proportional to the maximal slope) of the magnetic signal. PW50 indicates the half width of this function. (TIF) [file pone.0025834.s002.tif]
